# Dementia is associated with medial temporal atrophy even after accounting for neuropathologies

**DOI:** 10.1093/braincomms/fcac052

**Published:** 2022-03-07

**Authors:** Davis C. Woodworth, Nasim Sheikh-Bahaei, Kiana A. Scambray, Michael J. Phelan, Mari Perez-Rosendahl, María M. Corrada, Claudia H. Kawas, Seyed Ahmad Sajjadi, for the Alzheimer's Disease Neuroimaging Initiative

**Affiliations:** 1 Department of Neurology, University of California, Irvine, CA, USA; 2 Institute for Memory Impairments and Neurological Disorders, University of California, Irvine, CA, USA; 3 Department of Radiology, University of Southern California, Los Angeles, CA, USA; 4 Department of Pathology and Laboratory Medicine, University of California, Irvine, CA, USA; 5 Department of Epidemiology, University of California, Irvine, CA, USA; 6 Department of Neurobiology and Behavior, University of California, Irvine, CA, USA

**Keywords:** dementia, MRI, medial temporal lobe, atrophy, neuropathology

## Abstract

Brain atrophy is associated with degenerative neuropathologies and the clinical status of dementia. Whether dementia is associated with atrophy independent of neuropathologies is not known. In this study, we examined the pattern of atrophy associated with dementia while accounting for the most common dementia-related neuropathologies. We used data from National Alzheimer’s Coordinating Center (*n* = 129) and Alzheimer’s Disease Neuroimaging Initiative (*n* = 47) participants with suitable *in vivo* 3D-T_1_w MRI and autopsy data. We determined dementia status at the visit closest to MRI. We examined the following dichotomized neuropathological variables: Alzheimer’s disease neuropathology, hippocampal sclerosis, Lewy bodies, cerebral amyloid angiopathy and atherosclerosis. Voxel-based morphometry identified areas associated with dementia after accounting for neuropathologies. Identified regions of interest were further analysed. We used multiple linear regression models adjusted for neuropathologies and demographic variables. We also examined models with dementia and Clinical Dementia Rating sum of the boxes as the outcome and explored the potential mediating effect of medial temporal lobe structure volumes on the relationship between pathology and cognition. We found strong associations for dementia with volumes of the hippocampus, amygdala and parahippocampus (semi-partial correlations ≥ 0.28, *P* < 0.0001 for all regions in National Alzheimer’s Coordinating Center; semi-partial correlations ≥ 0.35, *P* ≤ 0.01 for hippocampus and parahippocampus in Alzheimer’s Disease Neuroimaging Initiative). Dementia status accounted for more unique variance in atrophy in these structures (∼8%) compared with neuropathological variables; the only exception was hippocampal sclerosis which accounted for more variance in hippocampal atrophy (10%). We also found that the volumes of the medial temporal lobe structures contributed towards explaining the variance in Clinical Dementia Rating sum of the boxes (ranging from 5% to 9%) independent of neuropathologies and partially mediated the association between Alzheimer’s disease neuropathology and cognition. Even after accounting for the most common neuropathologies, dementia still had among the strongest associations with atrophy of medial temporal lobe structures. This suggests that atrophy of the medial temporal lobe is most related to the clinical status of dementia rather than Alzheimer's disease or other neuropathologies, with the potential exception of hippocampal sclerosis.

## Teaser

Woodworth *et al*. report that MRI-measured atrophy of the medial temporal lobe is strongly associated with dementia even after accounting for brain pathologies from an autopsy. This suggests atrophy seen on MRI is reflective of neurodegeneration not entirely accounted for by common pathologies including Alzheimer’s disease.

## Graphical Abstract

**Graphical Abstract:**
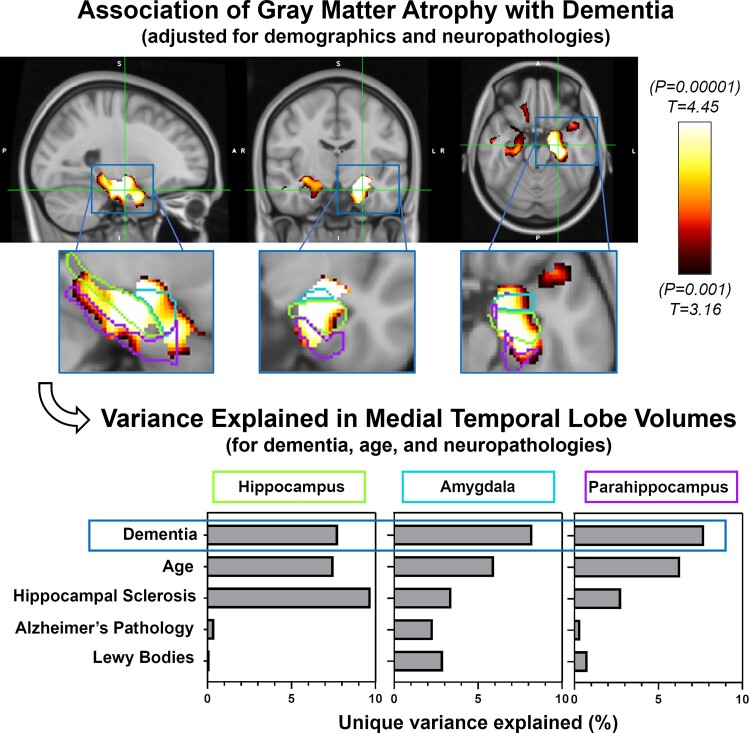

## Introduction

Some of the earliest imaging biomarkers proposed for Alzheimer’s disease were measures of brain atrophy. In 1988, Seab *et al.*^1^ reported a 40% reduction in hippocampal volume in Alzheimer’s disease patients compared with control participants. In 1989, de Leon *et al.*^2^ found that in participants with symptoms of dementia, hippocampal atrophy, as assessed on CT images, was more prevalent. Since then, many studies have found associations between atrophy of brain regions and clinical Alzheimer’s disease, including some recurring regions such as structures of the medial temporal, lateral temporal and parietal lobes, and these atrophy measures have been used for both diagnosis^3^ and as research biomarkers^4^ for Alzheimer’s disease.

Previous studies have also examined the association between brain atrophy, as assessed on imaging, and various neurodegenerative pathologies. An early study found that MRI- and autopsy-measured atrophy of the hippocampus were highly correlated and associated with lower hippocampal neuron counts.^5^ Another early imaging-pathology correlation study, using participants from the Nun Study, found that hippocampal atrophy was significantly associated with Braak staging of neurofibrillary tangles.^6^ Additional studies have found associations between Alzheimer’s disease neuropathology (ADNP) and whole brain, as well as hippocampal atrophy, while other neuropathologies, such as hippocampal sclerosis of aging and transactive response DNA-binding protein 43 kDa (TDP-43), have also been found to be associated with hippocampal volumes and medial temporal lobe atrophy.^7^

Only a few studies have examined the association between atrophy on neuroimaging and clinical dementia while accounting for neuropathologies. The objective of this study was to examine the association between MRI-measured antemortem grey matter (GM) atrophy and the clinical status of dementia while accounting for commonly assessed neuropathologies found at autopsy. To address this question, we used data from participants with appropriate MRI and neuropathology data from the National Alzheimer’s Coordinating Center (NACC) as an exploration data set and the Alzheimer’s Disease Neuroimaging Initiative (ADNI) database as a validation data set. We first examined associations between atrophy from MRI scans and cognition while accounting for pathologies, representing a clinician’s perspective of examining atrophy on a scan as the outcome in relation to clinical presentation and potential underlying pathologies. Then, we examined the added contribution of brain atrophy to demographic and neuropathological variables in relation to cognition as the outcome. We also examined whether brain atrophy mediated the effect of neuropathologies on cognition to explore potential causal mechanisms.

## Materials and methods

### Exploration data set, NACC

We used data from participants with and without dementia, who had both MRI and pathology data available in the NACC database between September 2005 and March 2017. The NACC Uniform Data Set (UDS) consists of data submitted by ∼30 National Institute on Aging (NIA) funded Alzheimer’s Disease Research Centers across the USA.^8^ Contributing Alzheimer’s Disease Research Centers are approved by their local institutional review board. We identified 243 participants with both pathology data and at least one available MRI and used 129 for our analyses (see MRI section below).

#### Demographic and clinical variables, NACC data

We selected dementia status, assigned by Alzheimer’s Disease Research Center clinicians as either dementia of the Alzheimer’s type or other non-Alzheimer’s dementia, at the assessment closest to MRI as our variable of interest. We also selected the following demographic variables: sex, age at MRI, years from MRI to death and years of education. We used mini-mental state examination (MMSE) and Clinical Dementia Rating sum of boxes (CDR-SBs) instead of dementia status for some analyses. We also used apolipoprotein E (APOE) information, dichotomized by the presence of any e4 allele, for a sub-analysis.

#### Pathology

Neuropathological data are collected at the Alzheimer’s Disease Research Centers via a standardized Neuropathology Form and Coding Guidebook.^9,10^ The NACC pathology data dictionary was used to define pathological categories. We assessed ADNP, which we defined as present (+) based on high likelihood per the NIA-Reagan criteria,^11^ comprising a combined Consortium to Establish a Registry for Alzheimer’s Disease (CERAD) neuritic plaque score of 3 (Frequent) and high Braak stage for neurofibrillary tangles (V/VI). We assessed hippocampal sclerosis, defined as present/absent (+/−) based on either NPHIPSCL (NACC pathology form version 10), categorizing hippocampal sclerosis as present or absent (+/−) in the CA1 and/or subiculum, and NPSCL (NACC pathology form version 9 and earlier), categorizing medial temporal lobe sclerosis as present or absent (+/−) including hippocampal sclerosis. We also assessed cerebral amyloid angiopathy (CAA) defined as none/mild (−) and moderate/severe (+); Lewy bodies, which we dichotomized as present/absent (+/−) based on presence in any region assessed; and atherosclerosis defined as none/mild (−) and moderate/severe (+). We excluded arteriolosclerosis from our primary analyses due to more limited availability in the NACC data used (76%). We also opted to exclude discrete vascular lesions (gross infarcts and lacunes, microinfarcts, and haemorrhages and microbleeds) for two reasons: (i) these lesions occur somewhat randomly in various brain regions and thus are less suited for examination by voxel-based morphometry (VBM), and (ii) this would reduce the number of variables and thus help mitigate potential overfitting. When the above-listed vascular pathologies were added to analyses, none were significantly associated with GM atrophy in the medial temporal lobe (data not shown). There was limited availability of hippocampal TDP-43 information for the participants in the data freeze of NACC used for this study (23 of 129, or 18% of participants), and thus TDP-43 was not included for analyses. However, TDP-43 data were available for more ADNI participants and used for supplemental analysis (see ‘Validation data set, ADNI’ section below). We also used the full range of severity (for atherosclerosis and CAA) and staging (for Braak tangles and CERAD plaques) scores as continuous variables for some analyses.

#### MRI

Supplementary Figure 1 shows a flow chart for the inclusion and exclusion criteria of NACC participants. Of the 243 participants with both pathology and at least one MRI available, 29 did not have a 3D-T_1_w scan. We decided to limit the data to scans that used an inversion recovery (IR) preparation pulse because this has a large effect on tissue contrast, especially between GM and white matter (WM).^12,13^ There were no differences between participants with IR compared with non-IR sequences. In the NACC database, there is a wide variation in MRI scanner vendor, model, field strength and acquisition parameters for 3D-T_1_w scans. IR sequences comprised IR fast spoiled gradient recalled echo on general electric (GE) scanners and magnetization-prepared rapid acquisition gradient echo (MPRAGE) on GE or Siemens scanners. We selected these anticipating that they would present with improved GM/WM contrast compared to non-IR pulse sequences. This was verified by visual assessment (data not shown) and the finding that, on average, non-IR scans presented with 7% less GM than the IR scans (*P* < 0.001, Student’s *t*-test). There were no differences between participants with and without IR scans in terms of the number of participants with dementia (*P* = 0.7), ADNP (*P* = 0.8), hippocampal sclerosis (*P* = 0.7, *χ*^2^ tests), or in terms of age at MRI (*P* = 0.4) or years from MRI to death (*P* = 0.14, *t*-tests). In addition, since ADNI data consist of IR scans only, limiting the NACC data to IR scans allowed for a better comparison between the data sets. Restricting the scans to those with IR sequences excluded another 74 participants rendering a sample of 140 participants with appropriate scans. To reduce the time from scan to autopsy, for each participant, we used the last available scan of sufficient quality.

The 3D-T_1_w IR scans were processed using the Computational Anatomy Toolbox (CAT12, http://www.neuro.uni-jena.de/cat/),^14^ which is implemented in the Statistical Parametric Mapping (SPM12, https://www.fil.ion.ucl.ac.uk/spm/software/spm12/) software. Preprocessing involved normalization, bias correction and skull-stripping of the T_1_w images followed by tissue segmentation into GM, WM, CSF and WM hypointensities based on the SPM12 tissue probability maps. Five participants failed segmentation: one participant was missing a substantial portion of brain tissue in the left lateral temporal lobe, and four participants had incorrect tissue class segmentation. Participants that failed processing were excluded from further analyses. An additional six participants were excluded due to missing data on one or more pathological variables. This left 129 participants as the final cohort used for analyses. For VBM analyses, the GM density images were realigned (normalized) to a common space, the voxel density values multiplied by the determinant of the Jacobian from normalization (modulated), smoothed with a Gaussian filter with a full-width at half maximum of 8 mm and thresholded at a GM density of 0.05. For region of interest (ROI) analyses, the unsmoothed but modulated and normalized GM density images were used, and volumes were extracted from ROIs defined by labels from the Harvard–Oxford cortical and subcortical atlases (maximum probability thresholds of 25%). Total intracranial volumes (TIVs) were computed using CAT12. As a comparison to and validation for the CAT12 volume estimates, we also calculated hippocampal volumes using two other methods: FreeSurfer hippocampal subfield segmentation^15^ and Automatic Segmentation of Hippocampal Subfields (ASHSs) Penn Memory Center T_1_-Only Atlas for T_1_-weighted 3T MRI pipelines.^16^ All MRIs were 3D-T_1_w scans with roughly 1 mm^3^ isotropic resolution and full brain coverage, thus suitable for processing through both pipelines. For FreeSurfer segmentations, FreeSurfer v6.0 was used. First, standard FreeSurfer processing was performed through the *recon-all* command. Then, FreeSurfer hippocampal subfield segmentation module was run, which we chose because it tended to better exclude CSF, but volume estimates were similar to those from recon-all (data not shown). All segmentations were visually inspected for the correctness of hippocampal estimates (i.e. exclusion of surrounding cerebrospinal fluid, WM and brainstem). For scans that did not process correctly due to *recon-all* failure, alternative skullstrip or Talairach registration steps were performed. Volume estimates came from the segmentation files and addition of the whole hippocampal volumes for the left and right hemispheres. ASHS produces segmentation of medial temporal lobe structures, including the anterior and posterior hippocampus. Processing was performed using Nifti files converted to ITK-SNAP (http://www.itksnap.org/pmwiki/pmwiki.php) workspaces that were uploaded to the cloud utility offered by ASHS (https://sites.google.com/view/ashs-dox/cloud-ashs/cloud-ashs-for-t1-mri). The resulting segmentations were assessed for quality control of the anterior and posterior hippocampal regions. Thirty-two segmentations had excessive inclusion of the ventricle or choroid plexus: these segmentations were fixed manually by erasing the erroneously labelled voxels in each coronal plane where they appeared. Estimates of volumes were generated from the segmentation files by adding the anterior and posterior hippocampus for the left and right hemispheres. Ultimately, 120 of the 129 total participants had suitable segmentation using all three pipelines, and data from these 120 participants were used for correlation and multiple linear regression analyses comparing the volumes generated by the techniques.

### Validation data set, ADNI

To validate the results found in the NACC data set, we used data from ADNI-1 participants who had both neuroimaging and pathology data available (*n* = 47). ADNI was launched in 2003, led by Principal Investigator Michael W. Weiner, MD, and has acquired serial MRI, PET, other biological markers and clinical and neuropsychological assessment, to study the progression of mild cognitive impairment (MCI) and early Alzheimer’s disease. For up-to-date information, see www.adni-info.org. We selected ADNI-1 participants because the majority of participants with pathology data (47 of 64 in the April 2018 public release) were ADNI-1. In addition, the ADNI-1 MRI protocol consisted of an MPRAGE sequence on 1.5T scanners that renders the sequences more comparable across scanners. We used the clinical classification of Alzheimer’s disease according to ADNI criteria^17^ as our dementia variable. Briefly, physicians classified participants as Alzheimer’s disease based on clinical assessment, an MMSE score of <26, a clinical dementia rating (CDR) score of 0.5 or greater and meeting NINCDS/ADRDA criteria^18^ for probable Alzheimer’s disease. We used the MRI closest to death for our analyses. Because ADNI neuropathology data are acquired following the NACC guidelines and forms (version 10), we used the same neuropathological variables described in the ‘*Pathology’* Methods section above, with NPHIPSCL as the only variable for hippocampal sclerosis. For a supplemental analysis, we used TDP-43 in the hippocampus (NPTDPC variable) which was available for a larger proportion of participants in ADNI (43 of 47, 91%) than NACC (18%).

### Statistical analysis

First, we examined the outcome of GM volumes in relation to both cognition and neuropathologies found at autopsy to potentially inform clinical interpretation of findings on scans in relation to clinical information and potential underlying pathologies. To examine whether GM volume was associated with clinical status of dementia while accounting for neuropathologies, we performed a VBM analysis in the NACC data set using multiple linear regression models on a voxel-wise basis, with dementia status, dichotomized neuropathological variables, sex, age at MRI, years of education, years from MRI to death and TIV, as covariates. We performed this analysis in a mask of the entire GM (thresholded at a density of 0.05) using a voxel-wise threshold of *P* = 0.001 and a cluster size family-wise error rate threshold of *P* = 0.05. Based on the voxel-wise VBM results, we then explored the relationship between the volumes of the various ROIs, as the dependent variables, with the demographic and neuropathological variables via multiple linear regression models. To verify the findings using the dichotomized neuropathological variables, we performed a multiple linear regression in which we used severity scores for atherosclerosis and CAA as well as Braak stage and CERAD neuritic plaque scores as continuous variables. In addition, to verify that the findings with dementia were present for more continuous measures of cognition, we performed multiple linear regressions using scores of MMSE and CDR-SB instead of dementia status. To further mitigate some of the scan-related heterogeneity, we examined multiple linear regressions in the subset of participants with MPRAGE scans (*n* = 74), and those with a 3T MPRAGE scan (*n* = 54), to observe whether the relative contributions of dementia, demographic or neuropathological variables changed. For validation of the VBM-calculated hippocampal volumes, we performed Pearson’s correlations between these and those generated by FreeSurfer and ASHS, and also report semi-partial correlations for multiple linear regressions for hippocampal volumes generated by each of these techniques.

For validation, we performed multiple linear regressions of the ROIs in the ADNI data set. Since the publicly available ADNI data set is considerably smaller than the NACC, we used simplified statistical models by limiting the number of covariates to those found to be significantly associated (*P* < 0.05) with the volumes of the ROIs in the NACC data set. We compared the semi-partial correlation coefficients between the NACC and ADNI data sets as estimates of effect size and directionality. We next performed this same comparison except replacing the ADNP variable with Braak stage dichotomized as present for V/VI, as well as dichotomized by present for Braak stage of III/IV/V/VI. We examined whether semi-partial correlation coefficients differed when using TDP-43 instead of hippocampal sclerosis, or when using both TDP-43 and hippocampal sclerosis in the same multiple linear regression.

Finally, we examined the unique contribution of the ROI volumes to dementia status and clinical burden of dementia accounting for pathologies and demographic variables. We performed regression analyses using the NACC data with presence (dementia status) and burden (CDR-SB) of cognition as the outcome and the ROI volumes (normalized by TIV), neuropathological information and demographic data as independent variables. We selected pathologies that were significantly associated with cognition in the above models and performed mediation analyses using the *mediation* package in R,^19^ with separate models for each of the ROI volumes as the mediator, pathology as independent variables, and cognition (dementia status or CDR-SB) as the dependent variable and the remaining pathology and demographic variables as confounders. For the mediation analyses, we used non-parametric bootstrap estimations with 10 000 iterations and report the average direct effect of pathology on cognition, the average causal mediation effect of pathology on cognition through the ROI volumes and the proportion of the total effect of pathology on cognition that was mediated by the ROI volumes.

We report the square of the semi-partial correlation coefficient (sr) as a measure of the unique variance in the dependent variable explained by an additional independent variable. It is the change in the multiple *R*^2^ induced by the inclusion of an additional explanatory variable, after accounting for the contributions of all other independent variables in the model. This makes the semi-partial correlation and related variance explained more interpretable than partial correlations or standardized regression coefficients.^20^ All analyses were performed using *R* (v4.0.3).

### Data availability

NACC and ADNI data are freely available to researchers upon request.

## Results

### Participant characteristics, NACC

The characteristics of the NACC participants are summarized in Table 1. There were 129 participants, most of whom presented with dementia at the time of MRI (94, 73%). Participants with dementia were significantly younger at time of the MRI, were more likely to have ADNP, a higher Braak stage, higher CERAD neuritic plaque score, Lewy bodies and more severe CAA. There were no significant differences in terms of sex, APOE e4 status or years of education. Of note, around 75% of the participants without dementia had MCI at the time of MRI.

**Table 1:** Participant characteristics in the NACC data set

Variable	Participants without dementia (*N* = 35)	Participants with dementia (*N* = 94)	*χ* ^2^ or *t*-test *P*-value
Males	23 (66%)	66 (70%)	0.6
Age at MRI	82 (±9) years	75 (±9) years	<0.001
Years education	16 (±3) years	16 (±3) years	0.8
MCI	26 (74%)		
MMSE	26.7 (±2.6)	18.8 (±6.4)^a^	<0.001
CDR-SB	1.7 (±1.7)	7.7 (±4.2)	<0.001
APOE e4	5 (15%)^b^	15 (17%)^b^	0.8
Years MRI to death	4 (±2) years	3 (±2) years	0.08
ADNP	11 (31%)	61 (65%)	<0.001
Braak stage 0/I/II	4 (11%)	2 (2%)	0.02
Braak stage III/IV	8 (23%)	12 (13%)	
Braak stage V/VI	23 (66%)	80 (85%)	
CERAD plaque 0	2 (6%)	3 (3%)	0.02
CERAD plaque 1	2 (6%)	1 (1%)	
CERAD plaque 2	18 (51%)	27 (29%)	
CERAD plaque 3	13 (37%)	63 (67%)	
Hippocampal sclerosis	6 (17%)	16 (15%)	0.7
Lewy bodies	10 (29%)	49 (52%)	0.049
CAA	11 (31%)	64 (68%)	0.02
Atherosclerosis	14 (40%)	30 (32%)	0.3

Continuous variables (age at MRI, years from MRI to death and years of education) are reported as mean and standard deviations within each group and group differences are assessed by Student’s *t*-test. Categorical variables (sex, MCI, APOE e4 and pathological variables) are reported as number and percentage within each group and group differences are assessed by *χ*^2^ tests.

MCI, mild cognitive impairment; MMSE, mini-mental state examination; CDR-SB, Clinical Dementia Rating sum of boxes; APOE, apolipoprotein E; ADNP, Alzheimer’s disease neuropathology; CERAD, Consortium to Establish a Registry for Alzheimer’s Disease; CAA, cerebral amyloid angiopathy; Edu., education.

^a^ Five participants with dementia were missing MMSE score.

^b^ Five participants with dementia and one participant without dementia were missing APOE information.

### VBM voxel-wise results, NACC

**Figure 1:**
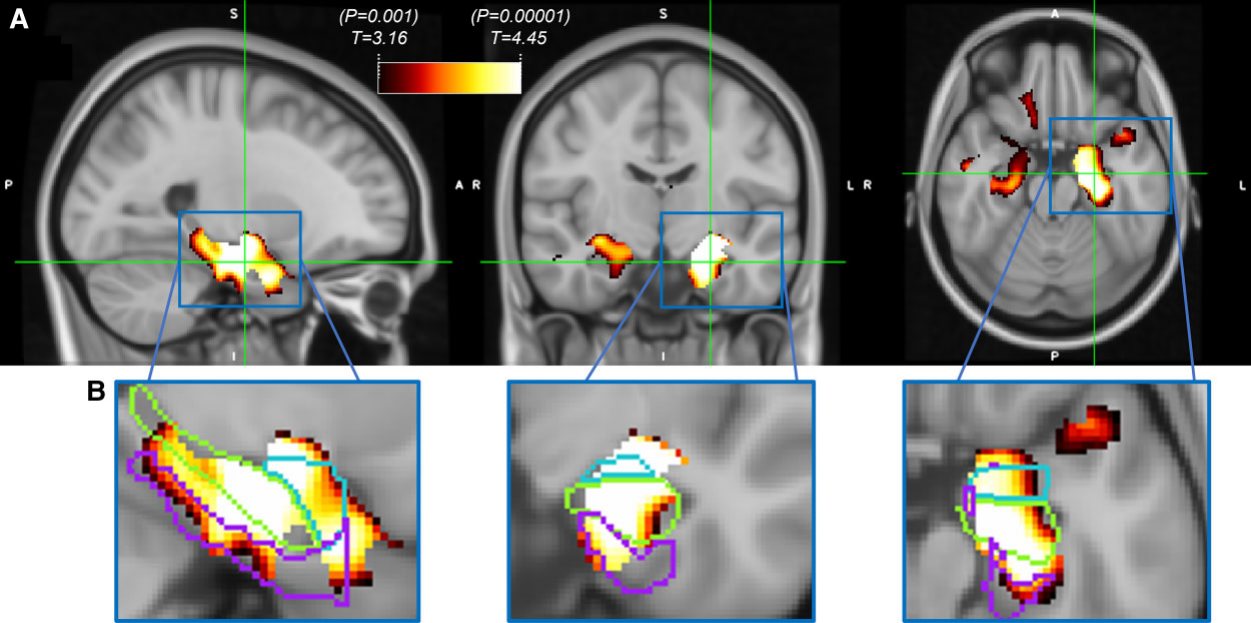
**Voxel-wise VBM association of decreased grey matter volume with dementia in NACC data (*N* = 129)**. Results using a mask of the entire grey matter, thresholded at *T*-stat = 3.16 (*P* = 0.001) and surviving a cluster-wise false discovery rate threshold of 0.05. (**A**) Strong associations present in the medial temporal lobe, with stronger associations in the left hemisphere. (**B**) Zoomed-in voxel-wise maps in left hemisphere with outlines of Harvard–Oxford atlas regions of the hippocampus (middle structure), amygdala (top structure) and parahippocampus (bottom structure) illustrating degree of overlap between thresholded *T*-statistic maps and these regions. Voxel-wise associations of grey matter volume with dementia adjusted for TIV, age, sex, education, years from MRI to death, ADNP, hippocampal sclerosis, CAA, Lewy bodies and atherosclerosis. VBM, voxel-based morphometry; TIV, total intracranial volume; ADNP, Alzheimer's disease neuropathology; CAA, cerebral amyloid angiopathy.

We observed strong associations between GM density and dementia in the hippocampus, amygdala and adjacent temporal cortex, even after accounting for other demographic and neuropathological variables, with stronger associations in the left hemisphere (left cluster size = 27.3 ml, right cluster size = 12.3 ml, cluster size family-wise error rate *P* < 0.001 for both; Fig. 1A). The bilateral Harvard–Oxford regions with the highest percentage overlap with the VBM results (thresholded at *P* = 0.001) were the amygdala (81%), hippocampus (64%), anterior parahippocampus (41%) and posterior parahippocampus (31%), Fig. 1B). The only other regions with significant overlap were the temporal pole (30%) and temporal fusiform gyrus (12%). We selected the medial temporal regions of the hippocampus, amygdala and parahippocampus (consisting of the anterior and posterior parahippocampal regions) as ROIs to be used for further analysis.

### ROI analysis, NACC

The volumes of the ROIs (hippocampus, amygdala, parahippocampus) all showed significant and strong negative associations with dementia status, and when examining the unique variance explained by each of the variables, we found that dementia status had the strongest association for all the regions, accounting for the most unique variance (∼8%, Table 2). The one exception to this was hippocampal sclerosis, which accounted for more variance of hippocampal volume (∼10%). In addition, we found significant associations between the volumes of these ROIs and age (greater atrophy with age), as well as TIV (greater intracranial volume associated with greater ROI volume). With regards to neuropathologies, the only significant associations with dementia were negative correlations between all ROIs and hippocampal sclerosis (strongest in the hippocampus with unique variance explained at ∼10%, lower in the other regions at ∼3%), and a negative correlation of amygdala volume with ADNP (2.3% variance explained) and Lewy bodies (2.9% variance explained). Adding dementia status to the models that already included the other variables increased the adjusted *R*^2^ from 0.34 to 0.42 for the hippocampus, 0.35 to 0.43 for amygdala and 0.27 to 0.35 for parahippocampus, and variance inflation factors for the models did not exceed 2 for any of the variables. Results were similar when using continuous instead of dichotomized measures for neuropathologies (Supplementary Table 1). Results for the sub-analysis adding APOE e4 information (Supplementary Table 2) revealed that presence of an e4 allele was significantly associated with amygdala volume (sr = −0.17, unique variance explained = 3%, *P* = 0.01), while for the hippocampus there was a trend (sr = −0.13, unique variance explained = 1.6%, *P* = 0.07) and no relationship was observed for the parahippocampus (sr = 0.01, unique variance explained = 0%, *P* = 0.9). Results were also similar when, instead of dementia, MMSE (hippocampus sr = 0.21, *P* = 0.004; amygdala sr = 0.21, *P* = 0.008; parahippocampus sr = 0.29, *P* < 0.001) and CDR-SB (hippocampus sr = −0.22, *P* = 0.001; amygdala sr = −0.24, *P* < 0.001; parahippocampus sr = −0.34, *P* < 0.001) were used in the model. Although dementia status did appear to have a slightly stronger association (hippocampus sr = −0.28, amygdala sr = −0.29, parahippocampus sr = −0.28); associations for other demographic and neuropathological variables were not substantially different when using CDR-SB or MMSE (data not shown). When limiting the participants used for analysis to those with more homogeneous scans (MPRAGE and 3T MPRAGE), the association of dementia with atrophy tended to increase, the association of hippocampal sclerosis with atrophy tended to increase as well, and the association of ADNP decreased in all regions and was no longer significant for the amygdala (Supplementary Table 3). As validation, the VBM-generated hippocampal volume showed a high correlation with FreeSurfer (*r* = 0.913, *P* < 0.001) and ASHS (*r* = 0.903, *P* < 0.001) hippocampal volumes, and the multiple linear regressions using each of the different measures yielded similar patterns confirming the observed results, although the effect sizes of variables and *R*^2^ were slightly reduced for the ASHS-generated hippocampal volumes (Supplementary Table 4).

**Table 2:** Multiple linear regression results for ROI volumes across the various demographic and neuropathological measures for the NACC data (*N* = 129)

Regions	Hippocampus				Amygdala				Parahippocampus			
Variables	Semi-Part.	% Var. Exp.	*T*-stat	*P*-value	Semi-Part.	% Var. Exp.	*T*-stat	*P*-value	Semi-Part.	% Var. Exp.	*T*-stat	*P*-value
*Demographic*												
Dementia status at MRI	**−0.28**	**7.78**	**−4.15**	**<0.001**	**−0.29**	**8.24**	**−4.30**	**<0.001**	**−0.28**	**7.73**	**−3.91**	**<0.001**
Age	**−0.27**	**7.51**	**−4.08**	**<0.001**	**−0.24**	**5.95**	**−3.65**	**<0.001**	**−0.25**	**6.30**	**−3.52**	**<0.001**
Sex	0.05	0.20	0.67	0.5	−0.04	0.18	−0.64	0.5	−0.12	1.35	−1.62	0.1
Years education	0.13	1.59	1.88	0.06	0.12	1.42	1.78	0.08	0.04	0.12	0.49	0.6
Years MRI to death	0.11	1.25	1.67	0.1	0.09	0.88	1.41	0.2	0.11	1.10	1.48	0.1
TIV	**0.19**	**3.61**	**2.84**	**0.005**	**0.19**	**3.50**	**2.80**	**0.006**	**0.15**	**2.28**	**2.13**	**0.04**
*Pathology*												
ADNP	−0.06	0.41	−0.96	0.3	−**0.15**	**2.31**	−**2.27**	**0.03**	−0.06	0.35	−0.83	0.4
Hippocampal sclerosis	−**0.31**	**9.73**	−**4.65**	**<0.001**	−**0.19**	**3.42**	−**2.77**	**0.006**	−**0.17**	**2.79**	−**2.35**	**0.02**
Lewy bodies	−0.04	0.13	−0.54	0.6	−**0.17**	**2.92**	−**2.56**	**0.01**	−0.09	0.79	−1.25	0.2
CAA	−0.03	0.08	−0.44	0.7	0.12	1.46	1.81	0.07	0.05	0.23	0.68	0.5
Atherosclerosis	−0.07	0.44	−0.98	0.3	−0.08	0.67	−1.22	0.2	−0.08	0.59	−1.08	0.3
*R* ^2^	0.47				0.48				0.41			

Bold denotes *P* < 0.05.

TIV, total intracranial volume; ADNP, Alzheimer’s disease neuropathology; CAA, cerebral amyloid angiopathy; Semi-Part., semi-partial correlation coefficient; Var. Exp., unique variance explained.

### ROI analysis, comparison of NACC and ADNI

For the simplified statistical model to compare the NACC and ADNI data, we only used the following variables which were significant in the models using the NACC data: dementia, age, TIV, ADNP, hippocampal sclerosis and Lewy bodies. Table 3 displays the participant characteristics for the ADNI data, which were broadly similar to those of the NACC data set. Dementia was significantly associated with lower volumes of the hippocampus and parahippocampus but not the amygdala, where there was only a trend and relatively similar semi-partial correlations among dementia, hippocampal sclerosis, ADNP and Lewy bodies (Table 4). The directionality of the findings for the neuropathological variables (ADNP and hippocampal sclerosis) were consistent for the ADNI and NACC data sets and broadly showed a similar magnitude, although ADNP had a larger (though not statistically significant) semi-partial correlation for each of the regions in the ADNI data set. When using dichotomized Braak stage instead of ADNP, the trends were largely similar, with nearly identical findings when using Braak stage V/VI or Braak stage III/IV/V/VI classifications (Supplementary Table 5). Finally, the association of dementia did not change when using TDP-43 instead of hippocampal sclerosis or when using both in the same model (Supplementary Table 6). TDP-43 tended to show similar associations when used instead of hippocampal sclerosis but the semi-partial correlations for TDP-43 and adjusted *R*^2^ for the model were lower, and when including both TDP-43 and hippocampal sclerosis in the same model hippocampal sclerosis tended to eclipse the associations of TDP-43.

**Table 3:** Participant characteristics in the ADNI data set

Variable	Participants without dementia (*N* = 11)	Participants with dementia (*N* = 36)	*χ* ^2^ or *t*-test *P*-value
Males	9 (82%)	24 (67%)	0.8
Age at MRI	84 (±4) years	80 (±6) years	0.1
MCI	9 (88%)	NA	
Years MRI to death	3 (±2) years	2 (±2) years	0.2
ADNP	5 (45%)	24 (67%)	0.2
Braak tangle stage 0/I/II	2 (18%)	9 (25%)	0.1
Braak tangle stage III/IV	2 (18%)	1 (3%)	
Braak tangle stage V/VI	7 (64%)	26 (72%)	
CERAD amyloid plaque 0	3 (27%)	5 (14%)	0.2
CERAD amyloid plaque 1	1 (9%)	6 (17%)	
CERAD amyloid plaque 2	2 (18%)	1 (3%)	
CERAD amyloid plaque 3	5 (45%)	24 (67%)	
Hippocampal sclerosis	2 (18%)	4 (11%)	0.5
Lewy bodies	5 (45%)	16 (44%)	0.6
TDP-43 Hipp.	3 (33%)^a^	14 (41%)^a^	0.7

Continuous variables (age at MRI, years from MRI to death) are reported as mean and standard deviation within each group and group differences are assessed by Student’s *t*-test. Categorical variables (sex and pathological variables) are reported as number and percentage within each group and group differences are assessed by *χ*^2^ tests.

MCI, mild cognitive impairment; ADNP, Alzheimer’s disease neuropathology; CERAD, Consortium to Establish a Registry for Alzheimer’s Disease; TDP-43, TAR DNA-binding protein 43; Hipp., hippocampus.

^a^ Two participants with and two participants without dementia were missing TDP-43 information in the hippocampus.

**Table 4:** Comparison of semi-partial correlation coefficients for ROI volumes across the various demographic and neuropathological measures in the NACC (*N* = 129) and ADNI (*N* = 47) data sets

Regions	Hippocampus semi-part. corr.		Amygdala semi-part. corr.		Parahippocampus semi-part. corr.	
Variables	NACC	ADNI	NACC	ADNI	NACC	ADNI
Dementia	−**0.34**	−**0.35**	−**0.30**	−0.21	−**0.31**	−**0.35**
Age	−**0.37**	0.11	−**0.34**	0.12	−**0.34**	0.11
TIV	**0.28**	0.23	**0.38**	0.19	**0.33**	0.09
ADNP	−0.05	−0.18	−0.15	−0.21	−0.05	−0.21
Hippocampal sclerosis	−**0.28**	−**0.47**	−**0.18**	−0.28	−**0.17**	−**0.35**
Lewy bodies	−0.05	−**0.29**	−**0.17**	−0.27	−0.07	−0.22
*R* ^2^	0.43	0.43	0.44	0.23	0.38	0.34

Bold denotes *P* < 0.05.

ROI, region of interest; TIV, total intracranial volume; ADNP, Alzheimer’s disease neuropathology; Semi-Part., semi-partial correlation coefficient.

### Contributions of ROI volumes to cognition, NACC

**Figure 2:**
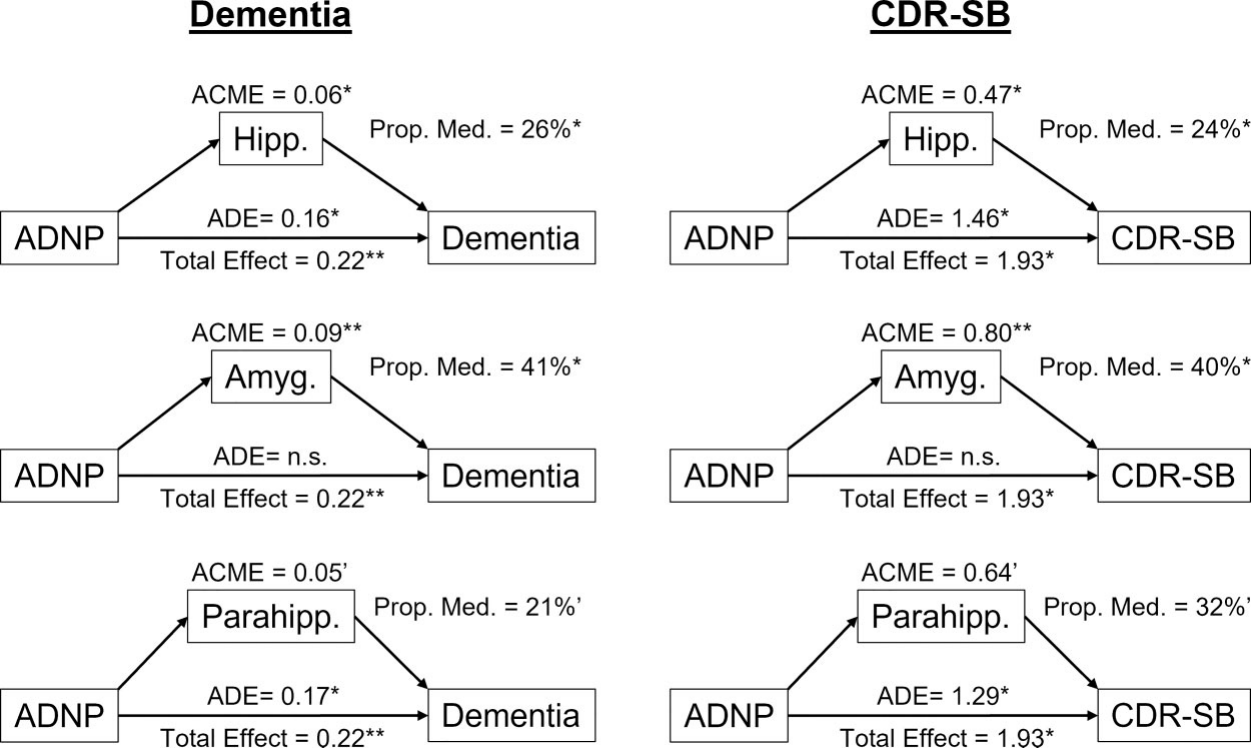
**Analyses for mediation of ROI volumes on the effect of ADNP on cognition**. Left column are mediation analyses with dementia status as the outcome, and right column are mediation analyses with CDR-SB as the outcome. Rows represent models with the volumes (normalized by TIV) as the mediators. Analyses adjusted for age, sex, education, years from MRI to death, hippocampal sclerosis, CAA, Lewy bodies and atherosclerosis. ADNP, Alzheimer's disease neuroathology; TIV, total intracranial volume; CAA, cerebral amyloid angiopathy; Hipp., hippocampus; Amyg., amygdala; Parahipp., parahippocampus; ACME, average causal mediation effect; ADE, average direct effect; Prop. Med., proportion mediated; ADNP, Alzheimer’s disease neuropathology; CDR-SB, Clinical Dementia Rating sum of the boxes. * *P* < 0.05. ** *P* < 0.01. ‘0.05 < *P* < 0.1.

In the logistic regression model using dementia as the outcome, the volumes of the regions were significant and strongly associated with dementia status (Table 5). Age, years until death and ADNP were also significant. In the multiple linear regressions with CDR-SB as the outcome, the medial temporal lobe ROIs were again significant and contributed to explaining the variance in CDR-SB, with hippocampal volume contributing 4.7%, amygdala volume contributing 5.4% and parahippocampal volume contributing 9.4% unique variance explained (Table 6). The contributions in unique variance explained by the ROI volumes to CDR-SB were the largest out of all the variables except for the interval between MRI to death, where participants with dementia were more likely to die sooner. Age and CAA were also significantly associated with CDR-SB, while ADNP was trending towards significance for the hippocampus (*P* = 0.058) and parahippocampus (*P* = 0.08). Finally, we chose the pathologies that were significant in the above analyses, ADNP and CAA, as the pathology variables of interest for our mediation analysis. While the relationship between CAA and cognition was not significantly mediated using any of the ROIs (data not shown), we found that the volumes for the hippocampus and amygdala partially mediated the relationship between ADNP and cognition (proportion mediated for dementia status was 26% for the hippocampus and 41% for the amygdala; while the proportion mediated for CDR-SB was 24% for the hippocampus and 40% for the amygdala, Fig. 2). Parahippocampal volume mediation only trended towards significance (proportion mediated for dementia, 21%; proportion mediated for CDR-SB, 32%).

**Table 5:** Results of logistic regression for dementia with models using either hippocampus, amygdala or parahippocampus volumes, alongside various demographic and neuropathological measures in the NACC data set (*N* = 129)

Regions	Outcome: dementia status								
	Model with hippocampus volume			Model with amygdala volume			Model with parahippocampus volume		
Variables	*β*	*Z*	*P*-value	*β*	*Z*	*P*-value	*β*	*Z*	*P*-value
*Demographic*									
Volume/TIV	−**1.24**	−**3.49**	**<0.001**	−**1.15**	−**3.51**	**<0.001**	−**0.98**	−**3.22**	**0.001**
Age	−**1.30**	−**3.10**	**0.002**	−**1.16**	−**2.90**	**0.004**	−**1.20**	−**2.99**	**0.003**
Sex	0.52	1.64	0.1	0.32	1.05	0.3	0.34	1.12	0.3
Years education	0.29	0.97	0.3	0.24	0.83	0.4	0.09	0.29	0.8
Years MRI to death	−**0.73**	−**2.46**	**0.01**	−**0.70**	−**2.43**	**0.02**	−**0.71**	−**2.49**	**0.01**
*Pathology*									
ADNP	**0.65**	**2.28**	**0.02**	0.52	1.80	0.07	**0.67**	**2.41**	**0.02**
Hippocampal sclerosis	−0.33	−1.18	0.2	−0.05	−0.20	0.8	−0.08	−0.33	0.7
Lewy bodies	0.46	1.60	0.1	0.28	0.98	0.3	0.32	1.15	0.3
CAA	0.36	1.36	0.2	**0.57**	**2.10**	**0.04**	0.45	1.69	0.09
Atherosclerosis	0.01	0.04	0.9	0.07	0.24	0.8	0.06	0.21	0.8
McFadden *R*^2^	0.34			0.34			0.32		

Bold denotes *P* < 0.05.

TIV, total intracranial volume; ADNP, Alzheimer’s disease neuropathology; CAA, cerebral amyloid angiopathy; Semi-Part., semi-partial correlation coefficient; Var. Exp., unique variance explained.

**Table 6:** Multiple linear regression results for CDR-SBs with models using either hippocampus, amygdala or parahippocampus volumes, alongside the various demographic and neuropathological measures for the NACC data (*N* = 129)

Regions	Outcome: CDR-SB											
	Model using hippocampus volume				Model using amygdala volume				Model using parahippocampus volume			
Variables	Semi-part.	% Var. exp.	*T*-stat	*P*-value	Semi-part.	% Var. exp.	*T*-stat	*P*-value	Semi-part.	% Var. exp.	*T*-stat	*P*-value
*Demographic*												
Volume/TIV	−**0.22**	**4.71**	−**2.89**	**0.005**	−**0.24**	**5.52**	−**3.14**	**0.002**	−**0.31**	**9.36**	−**4.23**	**<0.001**
Age	−**0.16**	**2.40**	−**2.07**	**0.04**	−**0.15**	**2.37**	−**2.06**	**0.04**	−**0.17**	**2.99**	−**2.38**	**0.02**
Sex	0.14	1.88	1.83	0.07	0.11	1.28	1.51	0.1	0.11	1.19	1.50	0.1
Years education	−0.04	0.13	−0.48	0.6	−0.04	0.14	−0.49	0.6	−0.08	0.56	−1.04	0.3
Years MRI to death	−**0.32**	**10.24**	−**4.26**	**<0.001**	−**0.32**	**10.50**	−**4.34**	**<0.001**	−**0.31**	**9.73**	−**4.30**	**<0.001**
*Pathology*												
ADNP	0.14	2.07	1.91	0.058	0.11	1.14	1.43	0.2	0.13	1.61	1.76	0.08
Hippocampal sclerosis	0.03	0.07	0.35	0.7	0.06	0.31	0.75	0.5	0.05	0.21	0.63	0.5
Lewy bodies	0.01	0.01	0.15	0.9	−0.03	0.09	−0.40	0.7	−0.02	0.03	−0.25	0.9
CAA	**0.18**	**3.28**	**2.41**	**0.02**	**0.22**	**4.97**	**2.98**	**0.003**	**0.19**	**3.57**	**2.61**	**0.01**
Atherosclerosis	−0.06	0.37	−0.82	0.4	−0.07	0.45	−0.89	0.4	−0.07	0.45	−0.92	0.4
*R* ^2^	0.33				0.34				0.38			

Bold denotes *P* < 0.05.

CDR-SB, Clinical Dementia Rating sum of the boxes; TIV, total intracranial volume; ADNP, Alzheimer’s disease neuropathology; CAA, cerebral amyloid angiopathy; Semi-Part., semi-partial correlation coefficient; Var. Exp., unique variance explained.

## Discussion

We examined the relationship between GM atrophy and clinical dementia status while accounting for other demographic and commonly assessed neuropathological variables using the NACC and ADNI databases. We found a strong association between dementia and atrophy of medial temporal lobe structures, namely the hippocampus, amygdala and parahippocampus, even after accounting for neuropathologies. This association of medial temporal lobe atrophy with dementia was stronger than for any other demographic or neuropathological variables, except for hippocampal sclerosis in relation to hippocampal volume. We found similar direction and effect size for the association of dementia with atrophy for (i) models using both dichotomized and continuous measures for neuropathologies, (ii) both dementia status and more continuous measures of cognitive burden (MMSE and CDR-SB), (iii) hippocampal volumes generated from three different pipelines (CAT12, FreeSurfer, ASHS), (iv) subsets of participants with more homogeneous MRI acquisitions and (v) across two different imaging-pathology databases (NACC and ADNI). In addition, measures of medial temporal lobe atrophy significantly contributed towards explaining impaired cognition and partially mediated the relationship between ADNP and cognition. The consistency in the results across these methodological variations underscores the strength and robustness of our findings. The clinical implication of this finding is that medial temporal lobe atrophy should not necessarily be construed as a surrogate marker for degenerative pathologies (with the potential exception of hippocampal sclerosis) but rather as a more general indicator of neuronal damage and loss that can be caused by a variety of known, and other potentially unknown, factors.

Although the observed association of the clinical status of dementia and medial temporal lobe atrophy is not surprising and was previously reported by many other groups, we found that this association remains after accounting for neuropathologies. Perhaps surprisingly, we found the association of dementia with medial temporal lobe atrophy was stronger than that of most of the neuropathologies. Some studies using *in vivo* biomarkers for Alzheimer’s pathology have found similar results, such as stronger associations between hippocampal volume and cognition compared with CSF amyloid,^21^ neurodegeneration measured by 18-F-fluorodeoxyglucose (FDG) PET being a stronger predictor of cognition than amyloid PET^22^ and atrophy attenuating the effects of CSF amyloid and tau on cognition.^23^ These *in vivo* measures of Alzheimer’s disease offer important information during life. However, in our study, we used not *in vivo* biomarkers of pathology but the neuropathological ratings assigned at autopsy (including pathologies other than Alzheimer’s) which are considered the gold standard.

Although many studies have examined the relationship between brain atrophy and either cognitive status or neuropathological findings separately, far fewer studies have assessed these relationships in a contiguous fashion. Among studies that have assessed both aspects, there have been conflicting results, with some studies finding strong associations between atrophy and pathologies even after accounting for cognition,^6,24^ while other studies found an attenuated or no effect of Alzheimer’s disease pathology on atrophy when cognition was accounted for.^25–29^ In our analysis of the NACC data that included more participants than any of the previous studies, when dementia was accounted for, the presence of ADNP was either weakly or not significantly associated with the volumes of the medial temporal lobe structures. This was true when using ADNP or Braak stage, for different pipelines for segmenting the hippocampus, and across both NACC and ADNI data sets. Previous studies have suggested that neuronal death is a sufficient condition for an amnestic type syndrome typically associated with Alzheimer’s disease: a study by Ball *et al.* published in 1985 found that in participants with clinical Alzheimer’s disease, neuronal loss and gliosis were the only consistent findings.^30^ Our results seem to coincide with this train of thought, namely that neuronal death appears to be the cause of medial temporal lobe atrophy, but a high burden of ADNP does not necessarily result in this neuronal death and atrophy if there is no clinical presentation of dementia. A recent study using multiple linear regression models found that while ADNP explained 6% of hippocampal volume in the whole cohort, when split into participants with and participants without dementia the variance explained by ADNP dropped to 3% for each of these subgroups,^25^ suggesting that the effect of ADNP on the hippocampal atrophy was tightly linked to dementia. This study also found that the effect of hippocampal sclerosis and TDP-43 was more pronounced than that of ADNP and also found similar results when using dichotomized versus continuous versions of neuropathological variables, which are similar to the findings in our study.

A recent study examining hippocampal volumes on post-mortem MRI using Religious Orders Study (ROS) and Memory and Aging Project (MAP) data found that after accounting for demographic and neuropathological variables, the addition of hippocampal volumes to their models explained an additional 5% in the variance of cognitive decline in participants.^31^ In our analyses using cognition as the outcome, we found a similarly strong association of medial temporal lobe volumes with cognition and unique variance explained by these measures ranging from 5% to 9%. Thus, our results agree with the ROS/MAP study. In addition, we believe our study contributes the following: (i) we examined the contribution of dementia and neuropathologies to GM atrophy on brain MRI. Our study arguably provides a more clinical perspective from the standpoint of interpreting the common radiological finding of medial temporal atrophy. (ii) The MRIs used for our study were *in vivo*, reminiscent of the information clinicians acquire to help them predict the underlying explanation for cognitive impairment, while the ROS/MAP study used post-mortem MRI. (iii) The MRIs used for our study spanned a wide variation in scanners and sequences, yet we still found strong associations that survived the potential noise introduced by the aforementioned heterogeneity. The ability to reproduce this finding with more heterogeneous data confirms its utility in a clinical setting.

Our results of associations between medial temporal lobe structures and hippocampal sclerosis of aging are also of interest. Many previous studies have noted that hippocampal sclerosis is associated with hippocampal atrophy,^32–34^ and one study has even noted associations outside the medial temporal lobe.^35^ In addition, other studies have found TDP-43, the proteinopathy signature of hippocampal sclerosis, to be associated with hippocampal^25^ and additional brain atrophy.^36^ However, it is unclear how the relationship between hippocampal sclerosis pathology and atrophy may change when accounting for cognitive status. We found a strong association of hippocampal sclerosis with hippocampal volume (accounting for ∼10% of variance), while also finding some associations with the amygdala and parahippocampal volume, even when accounting for dementia. This finding underscores the relevance of hippocampal volume as a potential biomarker for hippocampal sclerosis. In addition, our analyses in a subset of ADNI participants with TDP-43 information indicated that while TDP-43 behaves similar to hippocampal sclerosis, its effects are weaker. However, this analysis was in a smaller subset of participants and represents a limitation in this study: further research in larger data sets should examine medial temporal lobe atrophy in relation to cognition while accounting for more complete TDP-43 staging.

The results from the analyses with cognition as outcome reinforce and complement our findings outlined above namely, in models taking pathologies, demographic information and volumes from imaging into consideration, the volumes of the hippocampus, amygdala and parahippocampus were still strongly related to cognition near the time of MRI, to a greater degree than the pathologies. In addition, the volumes from the medial temporal lobe structures partially mediated the association between ADNP and cognition, with the amygdala volume mediating a higher proportion of this effect compared with the hippocampus. Thus, volumes from MRI significantly contributed towards explaining variance in cognition at the time of MRI above and beyond contributions from neuropathological staging from autopsy and partially mediated the effect of some pathologies on cognition. Future research utilizing longitudinal data from scans and cognitive assessments may be able to explain the temporal course of events more fully in relation to imaging, pathology and cognition.

One of the limitations of this study is the heterogeneity of the MRI data, specifically in the NACC data set. We did, however, limit the sequence paradigms used in the analysis to those that implemented an IR pulse, as it results in the greatest difference in tissue contrast. We also performed analyses limited to subsets of more homogeneous scans and the associations in these analyses tended to be slightly stronger and appeared to confirm the results in the full data set. In addition, the ADNI data set circumvented some of these confounding factors by implementing a similar sequence across different scan vendors, all at 1.5 T. Also, the similar strength of associations with cognitive status in both data sets (a more heterogeneous and a more homogeneous sample) highlights the robustness of our finding and the potential clinical application of the results. Another limitation to this study is the high proportion of participants with dementia and with high Braak stage and CERAD neuritic plaque scores in the NACC data freeze used. While the ADNI data set partially circumvented this issue, further studies should examine these relationships in data sets with a more balanced proportion of participants without dementia and lower Alzheimer’s pathology burden. However, this is a limitation present in many neuropathology data sets where a large proportion of participants have dementia and a high ADNP burden.

One major limitation of this study is the lack of quantitative measures of pathology, which may better capture pathological burden that leads to medial temporal lobe atrophy compared with the more qualitative staging systems used in this study. Recent research suggests that even at advanced Braak stages and Thal phases, there are variations in the degree of total tau and amyloid accumulation.^37^ Such variation might lead to the weakening of the associations reported in this study. It is noteworthy, however, that the pathological scoring system used in this study is based on the current consensus criteria used for pathological staging and classification of Alzheimer’s disease and related disorders (e.g. NIA-AA criteria^38^) and potentially highlight the need for more quantitative measures of pathological burden to more fully explain the variability in atrophy patterns. Additionally, while hippocampal sclerosis is a highly regionally specific pathological assessment of the CA1 and subiculum regions, the staging pathology measures such as ADNP have more limited regional specificity and thus are less tied to the medial temporal lobe specifically; however, medial temporal lobe atrophy on scans is often construed as ADNP, and the findings from our study caution against automatically adopting this interpretation. Another limitation of the study was the scarcity of fine-grain characterization of FTLD and other non-Alzheimer’s tauopathies, and therefore we could not study the unique contribution of these tau subtypes to hippocampal atrophy. One final limitation of the study is germane to most dementia research in that the participants used in this study are not necessarily a representative sample of the community, limiting generalizability.

In conclusion, we found a significant association between atrophy of medial temporal lobe structures, namely the hippocampus, amygdala and parahippocampus, and clinical status of dementia, even while accounting for many of the commonly assessed neuropathologies. This association of medial temporal lobe atrophy with dementia was stronger than any other demographic or neuropathological variable, with the exception of hippocampal sclerosis in relation to hippocampal volume. ADNP was not associated with hippocampal volume but was weakly associated with amygdala volume in our models. These findings have significant implications on the diagnostic utility of MRI in dementia clinics and confirm the significant pathology-independent relationship between dementia and atrophy of structures that are generally considered to be the hallmark of Alzheimer’s disease.

## Supplementary Material

fcac052_Supplementary_Data

## Abbreviations

ADNI =: Alzheimer’s Disease Neuroimaging Initiative
ADNP =: Alzheimer’s disease neuropathology
CDR-SBs =: Clinical Dementia Rating sum of the boxes
IR =: inversion recovery
MMSE =: mini-mental state examination
MPRAGE =: magnetization-prepared rapid acquisition gradient echo
NACC =: National Alzheimer’s Coordinating Center
ROI =: region of interest
TDP-43 =: transactive response DNA-binding protein 43 kDa
TIV =: total intracranial volume

## Notes

A complete listing of ADNI investigators can be found at: http://adni.loni.usc.edu/wp-content/uploads/how_to_apply/ADNI_Acknowledgement_List.pdf
