# Supplementary material for: Dementia is associated with medial temporal atrophy even after accounting for neuropathologies
Source: Brain Commun. 2022 Mar 7;4(2):fcac052. doi: 10.1093/braincomms/fcac052 (PMC8952251; doi:10.1093/braincomms/fcac052)
Supplement: fcac052_Supplementary_Data [file fcac052_supplementary_data.pdf]

Supplementary Figure 1. Flowchart of inclusion criteria for National Alzheimer's Coordinating Center (NACC) participants.

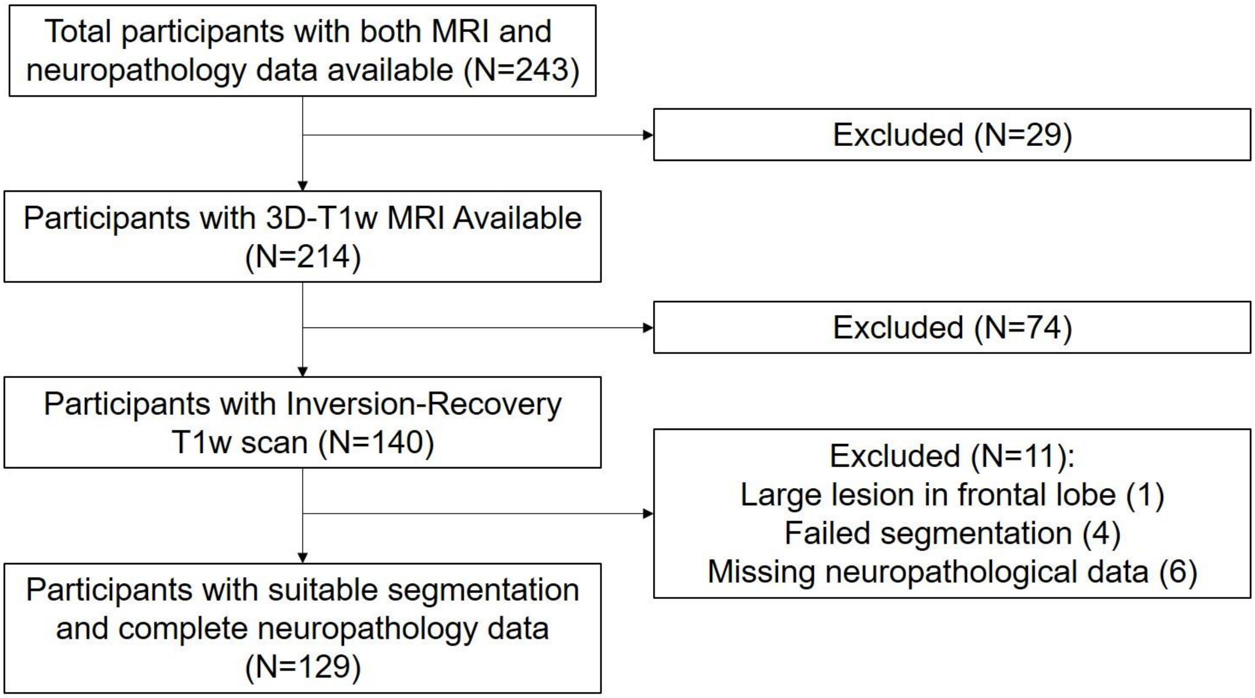

Supplementary Table 1. Multiple linear regression results for ROI volumes using full severity (CAA and atherosclerosis) or staging (Braak and CERAD) information as continuous variables in the NACC data (N=129).

	Regions	Hippocampus				Amygdala				Parahippocampus			
	**Variables**	**Semi-Part.**	**% Var. Exp.**	**T-stat**	**P-value**	**Semi-Part.**	**% Var. Exp.**	**T-stat**	**P-value**	**Semi-Part.**	**% Var. Exp.**	**T-stat**	**P-value**
Demographic	Dementia Status at MRI	**-0.29**	**8.29**	**-4.27**	**<0.001**	**-0.30**	**8.94**	**-4.40**	**<0.001**	**-0.27**	**7.29**	**-3.79**	**<0.001**
	Age	**-0.26**	**6.86**	**-3.89**	**<0.001**	**-0.25**	**6.20**	**-3.66**	**<0.001**	**-0.25**	**6.20**	**-3.50**	**<0.001**
	Sex	0.03	0.11	0.49	0.6	-0.03	0.07	-0.38	0.7	-0.13	1.74	-1.86	0.07
	Years Education	0.13	1.59	1.87	0.06	0.10	1.06	1.52	0.1	0.05	0.21	0.65	0.5
	Years MRI to Death	0.10	1.04	1.52	0.1	0.09	0.74	1.26	0.2	0.10	0.94	1.37	0.2
	TIV	**0.18**	**3.17**	**2.64**	**0.01**	**0.22**	**4.67**	**3.18**	**0.002**	0.13	1.72	1.85	0.07
Pathology	Braak Stage	-0.05	0.25	-0.74	0.5	0.02	0.03	0.23	0.8	-0.11	1.21	-1.55	0.1
	CERAD Plaques	0.02	0.03	0.24	0.8	-0.13	1.77	-1.96	0.053	0.04	0.16	0.57	0.6
	HS	**-0.30**	**8.70**	**-4.37**	**<0.001**	**-0.20**	**3.84**	**-2.89**	**0.005**	**-0.15**	**2.10**	**-2.04**	**0.044**
	Lewy Bodies	-0.04	0.13	-0.53	0.6	**-0.15**	**2.22**	**-2.19**	**0.03**	-0.09	0.76	-1.23	0.2
	CAA	-0.04	0.15	-0.59	0.6	0.09	0.83	1.34	0.2	0.05	0.29	0.77	0.4
	Atherosclerosis	-0.06	0.41	-0.95	0.3	-0.05	0.21	-0.67	0.5	-0.05	0.29	-0.77	0.4
	R^2	0.49				0.48				0.41			

Bold denotes $P < 0.05$. TIV: total intracranial volume. ADNP: Alzheimer's disease neuropathology. HS: Hippocampal Sclerosis. CERAD: Consortium to Establish a Registry for Alzheimer's Disease. CAA: Cerebral amyloid angiopathy. Semi-Part.: semi-partial correlation coefficient. Var. Exp.: unique variance explained.

Supplementary Table 2. Multiple linear regression results for ROI volumes across the various demographic and neuropathological measures, including APOE e4 status, for the NACC data (N=123).

	Regions	Hippocampus				Amygdala				Parahippocampus			
	**Variables**	**Semi-Part.**	**% Var. Exp.**	**T-stat**	**P-value**	**Semi-Part.**	**% Var. Exp.**	**T-stat**	**P-value**	**Semi-Part.**	**% Var. Exp.**	**T-stat**	**P-value**
Demographic	Dementia Status at MRI	**-0.29**	**8.24**	**-4.22**	**<0.001**	**-0.31**	**9.55**	**-4.61**	**<0.001**	**-0.27**	**7.29**	**-3.67**	**<0.001**
	APOE e4	-0.13	1.59	-1.86	0.07	**-0.17**	**3.03**	**-2.59**	**0.01**	0.01	0.01	0.13	0.9
	Age	**-0.30**	**8.82**	**-4.37**	**<0.001**	**-0.25**	**6.05**	**-3.67**	**<0.001**	**-0.26**	**6.55**	**-3.47**	**<0.001**
	Sex	0.03	0.10	0.47	0.6	-0.05	0.23	-0.72	0.45	-0.12	1.44	-1.62	0.1
	Years Education	0.13	1.72	1.93	0.056	0.13	1.56	1.86	0.07	0.03	0.08	0.38	0.7
	Years MRI to Death	0.11	1.17	1.59	0.1	0.09	0.74	1.28	0.2	0.09	0.81	1.22	0.2
	TIV	**0.17**	**2.96**	**2.52**	**0.01**	**0.18**	**3.20**	**2.67**	**0.009**	0.14	2.07	1.95	0.053
Pathology	ADNP	-0.07	0.48	-1.01	0.3	**-0.15**	**2.25**	**-2.24**	**0.03**	-0.05	0.28	-0.72	0.5
	HS	**-0.32**	**10.30**	**-4.72**	**<0.001**	**-0.19**	**3.69**	**-2.86**	**0.005**	**-0.16**	**2.56**	**-2.17**	**0.03**
	Lewy Bodies	-0.04	0.14	-0.55	0.6	**-0.14**	**2.07**	**-2.14**	**0.03**	-0.09	0.88	-1.28	0.2
	CAA	0.01	0.01	0.18	0.9	**0.18**	**3.06**	**2.60**	**0.01**	0.03	0.08	0.39	0.7
	Atherosclerosis	-0.05	0.25	-0.74	0.5	-0.07	0.53	-1.08	0.3	-0.07	0.52	-0.98	0.3
	R^2	0.49				0.51				0.40			

Bold denotes $P < 0.05$. TIV: total intracranial volume. ADNP: Alzheimer's disease neuropathology. HS: Hippocampal Sclerosis. CERAD: Consortium to Establish a Registry for Alzheimer's Disease. CAA: Cerebral amyloid angiopathy. Semi-Part.: semi-partial correlation coefficient. Var. Exp.: unique variance explained.

Supplementary Table 3. Semi-partial correlation coefficients for ROI volumes using progressively more homogeneous scan subsets.

	Regions	Hippocampus			Amygdala			Parahippocampus		
	**Variables**	**All IR (N=129)**	**MPRAGE (N=74)**	**MPRAGE 3T (N=54)**	**All IR (N=129)**	**MPRAGE (N=74)**	**MPRAGE 3T (N=54)**	**All IR (N=129)**	**MPRAGE (N=74)**	**MPRAGE 3T (N=54)**
Demographic	Dementia Status at MRI	**-0.28**	**-0.39**	**-0.35**	**-0.29**	**-0.34**	**-0.30**	**-0.28**	**-0.35**	**-0.29**
	Age	**-0.27**	**-0.23**	**-0.32**	**-0.24**	**-0.23**	**-0.26**	**-0.25**	**-0.24**	**-0.37**
	Sex	0.05	-0.02	-0.02	-0.04	-0.02	0.00	-0.12	-0.10	-0.02
	Years Education	0.13	0.17	**0.22**	0.12	0.09	0.11	0.04	0.09	0.12
	Years MRI to Death	0.11	0.08	0.04	0.09	0.02	-0.02	0.11	-0.01	-0.06
	TIV	**0.19**	**0.12**	0.10	**0.19**	**0.28**	**0.29**	**0.15**	**0.18**	**0.22**
Pathology	ADNP	-0.06	-0.01	-0.06	**-0.15**	-0.05	-0.06	-0.06	0.04	-0.02
	HS	**-0.31**	**-0.37**	**-0.40**	**-0.19**	**-0.23**	**-0.24**	**-0.17**	**-0.25**	**-0.26**
	Lewy Bodies	-0.04	0.03	0.06	**-0.17**	**-0.15**	-0.17	-0.09	-0.10	-0.08
	CAA	-0.03	-0.03	0.01	0.12	0.10	0.11	0.05	0.09	0.05
	Atherosclerosis	-0.07	-0.10	0.01	-0.08	-0.07	-0.01	-0.08	-0.10	0.03
	R^2	0.47	0.66	0.74	0.48	0.67	0.67	0.41	0.62	0.68

Bold denotes $P < 0.05$. TIV: total intracranial volume. ADNP: Alzheimer's disease neuropathology. HS: Hippocampal Sclerosis. CAA: Cerebral amyloid angiopathy. IR: inversion recovery. MPRAGE: magnetization-prepared rapid gradient echo.

Supplementary Table 4. Comparison of semi-partial correlation coefficients for hippocampal volumes estimated using different neuroimaging pipelines in NACC data (N=120).

		Hippocampus		
	**Variables**	**VBM**	**FS**	**ASHS**
Demographic	Dementia Status at MRI	**-0.28**	**-0.25**	**-0.21**
	Age	**-0.27**	**-0.23**	**-0.15**
	Sex	0.05	0.05	0.08
	Years Education	0.08	0.09	0.16
	Years MRI to Death	0.12	0.07	0.06
	TIV	**0.22**	**0.30**	**0.26**
Pathology	ADNP	-0.06	-0.07	-0.03
	HS	**-0.32**	**-0.27**	**-0.29**
	Lewy Bodies	-0.05	-0.01	-0.04
	CAA	-0.06	-0.01	-0.02
	Atherosclerosis	-0.06	-0.02	-0.06
R^2		0.45	0.45	0.37

Bold denotes P<0.05. TIV: total intracranial volume. ADNP: Alzheimer's Disease Neuropathology. HS: Hippocampal Sclerosis. CAA: Cerebral Amyloid Angiopathy. VBM: Voxel-Based Morphometry estimate from Computational Anatomy Toolbox (CAT12). FS: FreeSurfer Hippocampal Subfield Segmentation. ASHS: Automatic Segmentation of Hippocampal Subfields T1w.

Supplementary Table 5. Comparison of NACC (N=129) and ADNI (N=49) semi-partial correlation coefficients for ROI volumes across the various demographic and neuropathological measures, with dichotomized Braak stages instead of ADNP included in the model.

Dichotomized by Braak Stage V/VI	Hippocampus Semi-Part. Corr.		Amygdala Semi-Part. Corr.		Parahippocampus Semi-Part. Corr.	
**Variables**	**NACC**	**ADNI**	**NACC**	**ADNI**	**NACC**	**ADNI**
Dementia	**-0.35**	**-0.37**	**-0.33**	-0.23	**-0.31**	**-0.36**
Age	**-0.37**	0.11	**-0.32**	0.12	**-0.34**	0.11
TIV	**0.28**	0.21	**0.37**	0.15	**0.33**	0.05
Braak V/VI	-0.04	-0.17	-0.08	-0.25	-0.08	**-0.27**
HS	**-0.28**	**-0.46**	**-0.18**	-0.26	**-0.17**	**-0.33**
Lewy Bodies	-0.04	**-0.26**	**-0.16**	-0.24	-0.07	-0.20
R^2	0.43	0.43	0.42	0.26	0.38	0.37
**Dichotomized by Braak Stage III/IV**	**Hippocampus Semi-Part. Corr.**		**Amygdala Semi-Part. Corr.**		**Parahippocampus Semi-Part. Corr.**	
**Variables**	**NACC**	**ADNI**	**NACC**	**ADNI**	**NACC**	**ADNI**
Dementia	**-0.35**	**-0.39**	**-0.34**	-0.26	**-0.33**	**-0.39**
Age	**-0.37**	0.13	**-0.33**	0.15	**-0.34**	0.13
TIV	**0.28**	0.22	**0.37**	0.16	**0.33**	0.07
Braak III/IV/V/VI	-0.06	-0.10	-0.07	-0.21	-0.03	-0.20
HS	**-0.28**	**-0.47**	**-0.18**	**-0.28**	**-0.17**	**-0.35**
Lewy Bodies	-0.04	**-0.27**	**-0.16**	-0.26	-0.06	-0.21
R^2	0.43	0.41	0.42	0.24	0.38	0.33

Bold denotes $P < 0.05$. TIV: total intracranial volume. HS: Hippocampal Sclerosis. Semi-Part. Corr.: semi-partial correlation coefficient.

Supplementary Table 6. Comparison of semi-partial correlation coefficients for HS, TDP-43, or both variables in the same model, for ADNI data (N=43).

Regions	***Hippocampus Semi-Part. Corr.***			***Amygdala Semi-Part. Corr.***			***Parahippocampus Semi-Part. Corr.***		
Variables	***HS***	***TDP-43***	***Both***	***HS***	***TDP-43***	***Both***	***HS***	***TDP-43***	***Both***
Dementia	**-0.35**	-0.27	**-0.32**	-0.21	-0.16	-0.19	**-0.33**	-0.28	**-0.32**
Age	0.08	-0.01	0.07	0.18	0.13	0.17	0.15	0.09	0.15
TIV	0.23	0.19	0.24	0.20	0.18	0.21	0.11	0.07	0.11
ADNP	-0.15	-0.18	-0.17	-0.13	-0.16	-0.15	-0.15	-0.17	-0.16
HS	**-0.49**		**-0.41**	-0.29		-0.24	**-0.36**		**-0.31**
TDP-43 Hipp.		**-0.30**	-0.14		-0.20	-0.10		-0.20	-0.08
Lewy Bodies	**-0.26**	-0.14	**-0.26**	-0.28	-0.22	-0.28	-0.23	-0.14	-0.23
Adjusted R^2	**0.33**	0.15	**0.33**	0.12	0.06	0.10	**0.21**	0.11	**0.20**

Bold denotes P<0.05. TIV: total intracranial volume. ADNP: Alzheimer's Disease Neuropathology. HS: Hippocampal Sclerosis. Semi-Part. Corr.: semi-partial correlation coefficient. TDP-43: TAR DNA-binding protein 43. Hipp.: Hippocampus.
